# Development of an Immunogenomic Landscape-Based Prognostic Index of Head and Neck Squamous Cell Carcinoma

**DOI:** 10.3389/fmolb.2020.586344

**Published:** 2020-11-24

**Authors:** Jinhua Long, Shichao Zhang, Xianlin Zeng, Yan Ouyang, Yun Wang, Zuquan Hu, Yuannong Ye, Weili Wu, Feng Jin, Shi Zhou, Zhu Zeng

**Affiliations:** ^1^School of Basic Medical Sciences, Guizhou Medical University, Guiyang, China; ^2^School of Biology and Engineering, Guizhou Medical University, Guiyang, China; ^3^Key Laboratory of Infectious Immune and Antibody Engineering in Guizhou Province, Guizhou Medical University, Guiyang, China; ^4^Department of Head and Neck Oncology, The Affiliated Hospital of Guizhou Medical University, Guiyang, China; ^5^Key Laboratory of Biology and Medical Engineering, Immune Cells and Antibody Engineering Research Center of Guizhou Province, School of Biology and Engineering, Guizhou Medical University, Guiyang, China; ^6^Department of Intervention, The Affiliated Hospital of Guizhou Medical University, Guiyang, China; ^7^Key Laboratory of Environmental Pollution Monitoring and Disease Control, Ministry of Education, Guizhou Medical University, Guiyang, China

**Keywords:** head and neck squamous cell carcinoma, cancer immunology, bioinformatics, immunogenomic landscape, prognostic index

## Abstract

Head and neck squamous cell carcinoma (HNSCC) is the eighth leading cancer by incidence worldwide, with approximately 700,000 new cases in 2018 (accounting for 11% of all cancers). The occurrence and development of tumors are closely related to the immunological function of the body and sensitivity to treatment schemes as well as prognosis. It is urgent for clinicians to systematically study patients’ immune gene maps to help select a treatment plan and analyze the potential to cure HNSCC. Here, the transcriptomic data of HNSCC samples were downloaded from The Cancer Genome Atlas (TCGA), and 4,793 genes differentially expressed in normal and cancer tissues of HNSCC were identified, including 1,182 downregulated and 3,611 upregulated genes. From these genes, 400 differentially expressed immune-related genes (IRGs) were extracted, including 95 downregulated genes and 305 upregulated genes. The prognostic values of IRGs were evaluated by univariate Cox analysis, and 236 genes that were significantly related to the overall survival (OS) of patients were identified. The signaling pathways that play roles in the prognosis of IRGs were investigated by Gene Ontology (GO) and Kyoto Encyclopedia of Genes and Genomes (KEGG) analyses, and the expression profiles of IRGs and OS in 499 HNSCC patients based on TCGA dataset were integrated. Potential molecular mechanisms and characteristics of these HNSCC-specific IRGs were further explored with the help of a new prognostic index based on IRGs developed by least absolute shrinkage and selection operator (LASSO) Cox analysis. A total of 64 hub genes (IRGs associated with prognosis) were markedly associated with the clinical outcome of HNSCC patients. KEGG functional enrichment analysis revealed that these genes were actively involved in several pathways, e.g., cytokine–cytokine receptor interaction, T-cell receptor signaling, and natural killer cell-mediated cytotoxicity. IRG-based prognostic signatures performed moderately in prognostic predictions. Interestingly, the prognostic index based on IRGs reflected infiltration by several types of immune cells. These data screened several IRGs of clinical significance and revealed drivers of the immune repertoire, demonstrating the importance of a personalized IRG-based immune signature in the recognition, surveillance, and prognosis of HNSCC.

## Introduction

Head and neck squamous cell carcinoma (HNSCC) encompasses a heterogeneous group of epithelial malignancies that arise in the oral cavity, oropharynx, larynx, or hypopharynx (Cramer et al., 2019). Worldwide, HNSCC is the eighth leading cancer and accounts for over 700,000 new cancer cases and 350,000 deaths each year. Every year, 4–7% of patients with HNSCC develop distant metastasis, which is common in the head and neck, lung, and esophagus. Approximately half of newly diagnosed patients will not survive beyond 5 years. At diagnosis, 45% of patients already show regional lymph node metastasis (Leemans et al., 2018; Cramer et al., 2019). Moreover, the rate of distant metastasis in HNSCC patients is exceptionally high (Leemans et al., 2018). At present, the clinical treatments for HNSCC mainly include surgery, radiotherapy, chemotherapy, molecular targeting, and immunotherapy (Cramer et al., 2019). The formation of distant metastasis after surgery is one of the main reasons for the decline in the long-term survival rate of HNSCC (Cramer et al., 2019). The immune system modifications noted in HNSCC patients suggest that this cancer is an overall immunosuppressive process (Jin and Qin, 2020). In the peripheral bloodstream, HNSCC patients have a lower overall number of white blood cells, which comprise a greater proportion of suppressive regulatory T cells (Tregs) (Jin and Qin, 2020). Existing treatments are insufficient for patients with locally advanced or distantly metastatic HNSCC. Careful monitoring of the progression of HNSCC with the help of novel and sensitive biomarkers could reduce the number of HNSCC patients not diagnosed before the onset of aggressive disease.

In the middle of the last century, Burnt and Thomas proposed the theory of immune surveillance, which suggests that the immune system of the host can recognize precursors of cancer and, in most cases, destroy these precursors before they become clinically apparent (Li et al., 2020). After attack by the innate immune system, tumor antigens (TAs) are released and captured by antigen-presenting cells (APCs) and then processed and loaded onto the major histocompatibility complex (MHC), activating effector T cells. However, cancer cells can create an immunosuppressive microenvironment through manipulation of their own immunogenicity, production of immunosuppressive mediators, and promotion of immunomodulatory cell types, leading to immune tolerance and escape (Li et al., 2020). Therefore, immune normalization treatments and cancer immunotherapy have been major drivers of personalized medicine, with aggressive efforts to leverage the immune system to fight tumors (Sanmamed and Chen, 2018). The immune system has been recognized to be fundamental to the development, establishment, and spread of HNSCC. At present, several cutting-edge immunotherapies provide HNSCC patients with other alternative treatment protocols, e.g., monoclonal antibodies, immune checkpoint inhibitors (ICIs), and cell immunotherapy. Clinical trials show that patients with HNSCC can benefit from programmed cell death 1 (PD-1)/programmed cell death ligand 1 (PD-L1) therapy (one type of ICI) and obtain a better quality of life (Leemans et al., 2018; Cramer et al., 2019). There are plenty of immune cells in the microenvironment of HNSCC, but their functions are not well defined (Leemans et al., 2018). Furthermore, biomarkers that can accurately predict the response of patients with HNSCC to immunotherapy are still lacking, and their applications can help patients classify and select clinical treatment protocols (Cramer et al., 2019). Most recently, large-scale gene expression datasets enable cancer researchers to efficiently identify biomarkers for tumor monitoring and surveillance. Several researchers have comprehensively investigated the prognostic value of immune-related genes (IRGs) to build a personalized immune signature that can ameliorate prognostic estimations for patients with non-squamous non-small-cell lung cancer, papillary thyroid cancer, breast cancer, and renal cell carcinoma (Zhang S. C. et al., 2019; Zhang S. et al., 2019). IRGs play a vital role in the immune system that can control the immune response, including cytokine-related genes, chemokine-related genes, and other cell surface antigen genes. However, the clinical relevance and prognostic significance of IRGs in HNSCC are still elusive. In this study, IRG expression profiles were integrated with clinical information by computational methods for the evaluation of overall survival (OS) in HNSCC patients with the goal of gaining insight into the potential clinical application of IRGs in prognostic stratification and their implicational potential as biomarkers for targeted HNSCC therapy. On this basis, the expression status and prognostic landscape of IRGs were systematically analyzed, and an individualized prognostic signature for HNSCC patients was developed. The underlying molecular regulatory mechanisms were explored by bioinformatics. This study lays a theoretical foundation for further understanding the pathophysiological process and clinical individualized treatment of HNSCC.

## Materials and Methods

### Clinical Samples and Data Acquisition

Transcriptome RNA-sequencing data of 499 primary HNSCC and 44 non-tumor tissue samples were acquired from TCGA data portal^1^. Clinical information for these patients was obtained from the same source. Lists of IRGs were exported from the Immunology Database and Analysis Portal (ImmPort) database (Bhattacharya et al., 2014).

### Differential Gene Analysis

To filter IRGs involved in the incidence of HNSCC, differentially expressed IRGs between HNSCC and adjacent non-tumor tissue samples were screened *via* the Wilcoxon signed-rank test. False discovery rate (FDR) < 0.05 and log_2_ | fold change| > 1 were chosen as the cutoff values for differential gene analysis of all transcriptional data. Differentially expressed IRGs were then selected from all differentially expressed genes.

### Survival Analysis

Survival-associated IRGs were selected by univariate Cox analysis using R software survival package. Survival-related IRGs were also submitted for functional enrichment analysis.

### Molecular Characteristics of Hub Immune-Related Genes

Hub IRGs are differentially expressed IRGs that significantly correlated with clinical outcomes of HNSCC. Copy number alterations data were acquired from TCGA Copy Number Portal^2^ (Gao et al., 2013). To explore the interactions between hub IRGs, a protein–protein interaction (PPI) network was constructed based on the data gathered from the STRING online database^3^. The PPI network could visually display the direct or indirect interactions between hub IRGs. PPI results were visualized using Cytoscape (version 3.7.1) (He et al., 2018). To study the regulatory mechanisms of hub IRGs, regulatory links between potential transcription factors (TFs) and hub IRGs were built based on the Cistrome Cancer database. The Cistrome Cancer database stored cancer genomics data from TCGA along with over 23,000 ChIP-seq and chromatin accessibility profiles, which makes it an ideal tool for exploring the regulatory links between TFs and transcriptomes (Mei et al., 2017).

### Development of the Immune-Related Gene-Based Prognostic Index

Hub IRGs were submitted for least absolute shrinkage and selection operator (LASSO) Cox regression analyses, while integrated IRGs remaining as independent prognostic indicators for developing the immune-related gene-based prognostic index (IRGPI). Patient datasets were divided into high- and low-risk groups based on their median PI-value. The prognostic value of the PI was assessed in patients with different subtypes of HNSCC. The TIMER online database stored abundance information of tumor-infiltrating immune cells and provide useful interfaces for analyzing and visualizing them (Li et al., 2017). TIMER also reanalyzed gene expression data, with estimation of abundance of six subtypes of tumor-infiltrating immune cells, including B cells, CD4^+^ T cells, CD8^+^ T cells, macrophages, neutrophils, and dendritic cells (DCs) from 10,897 samples across 32 cancer types from TCGA. Therefore, it can be easily employed for determining the relationship between immune cell infiltration with cancer prognosis. In this study, the associations between immune infiltrate levels of HNSCC samples and their IRGPI level were calculated.

### Statistical Analysis

Gene functional enrichment analyses were conducted based on the R software cluster Profiler package for identifying biological themes among gene clusters (Yu et al., 2012). The area under the curve (AUC) of the survival receiver operating characteristic (ROC) curve was calculated using R software survival ROC package for validating the performance of the prognostic signature (Heagerty et al., 2000).

## Results

### Identification of Differentially Expressed Immune-Related Genes

**FIGURE 1:**
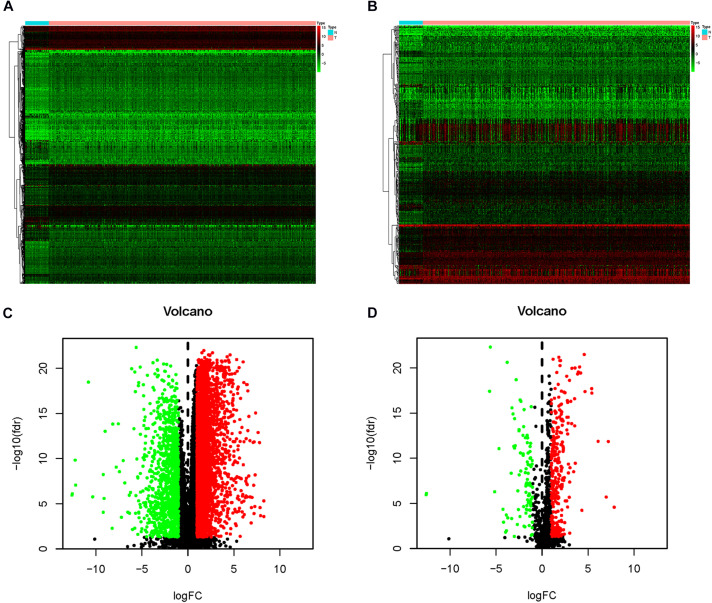
Differentially expressed immune-related genes (IRG). The heat map **(A)** and volcano map **(C)** indicate the differentially expressed genes between head and neck squamous cell carcinoma (HNSCC) tissues and normal tissues. Red dot indicates upregulated genes, green dot indicates downregulated genes, and black dot indicates genes without a difference. In the heat map **(B)** and volcano map **(D)**, differentially expressed IRGs are shown. The red dot indicates the highly expressed genes, the green dot indicates the downregulated genes, and the black dot indicates the genes with no difference.

The Wilcoxon signed-rank test identified 4,793 differentially expressed genes, of which 3,611 were upregulated and 1,182 were downregulated (Figures 1A,C). From this set of genes, 400 differentially expressed IRGs were extracted, including 305 upregulated and 95 downregulated genes (Figures 1B,D).

### Identification of Survival-Associated Immune-Related Genes

**FIGURE 2:**
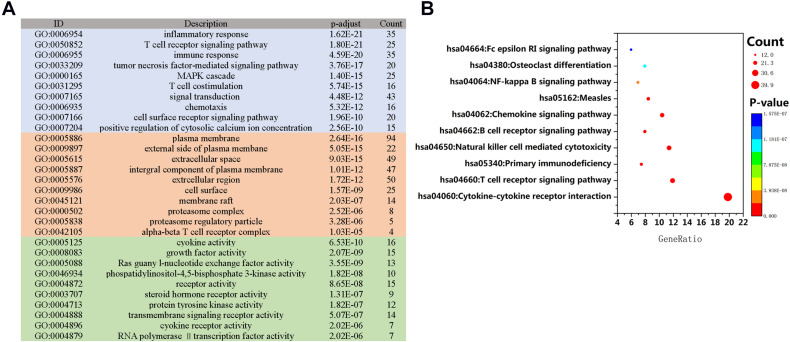
Gene function enrichment of immune-related genes related to survival. **(A)** Gene Ontology (GO) analysis; blue, red, and green bars represent biological processes, cellular components, and molecular functions, respectively. **(B)** The 10 most significant Kyoto Encyclopedia of Genes and Genomes (KEGG) signaling pathways.

Disease stage and classification are important bases for clinical decision-making and individualized therapy; therefore, one of the main targets of this study is to identify potential molecular biomarkers that could serve as significant clinical prognostic indicators. Univariate Cox analysis identified 236 IRGs that have significant correlations with OS in HNSCC patients. As predicted, gene functional enrichment analysis confirmed that the immune response was most frequently implicated. The most frequently appearing biological terms among biological processes, cellular components, and molecular functions are “inflammatory response,” “plasma membrane,” and “cytokine activity” (Figure 2A). Kyoto Encyclopedia of Genes and Genomes (KEGG) pathways showed that cytokine–cytokine receptor interactions were most often enriched by survival-associated IRGs (Figure 2B).

### Identification and Characteristics of Hub Immune Genes

**FIGURE 3:**
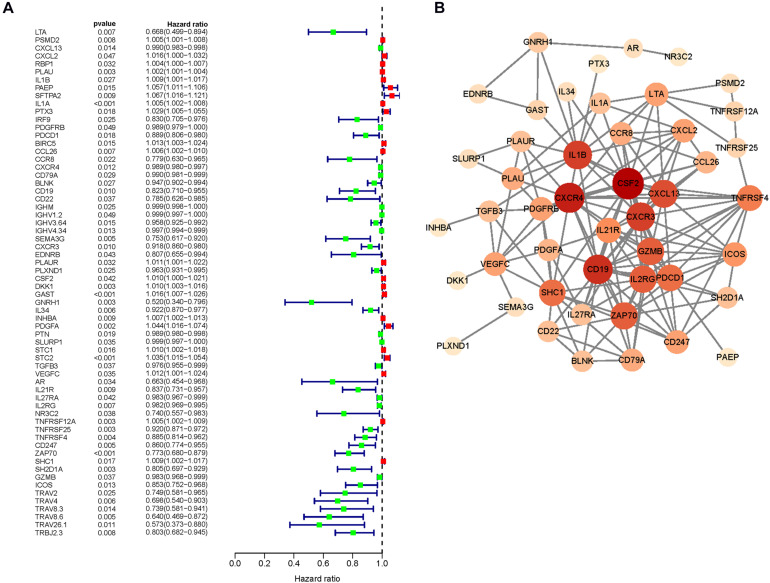
The prognostic value of core immune genes. **(A)** The forest map of hazard ratio (HR) value. **(B)** Protein interaction network.

**FIGURE 4:**
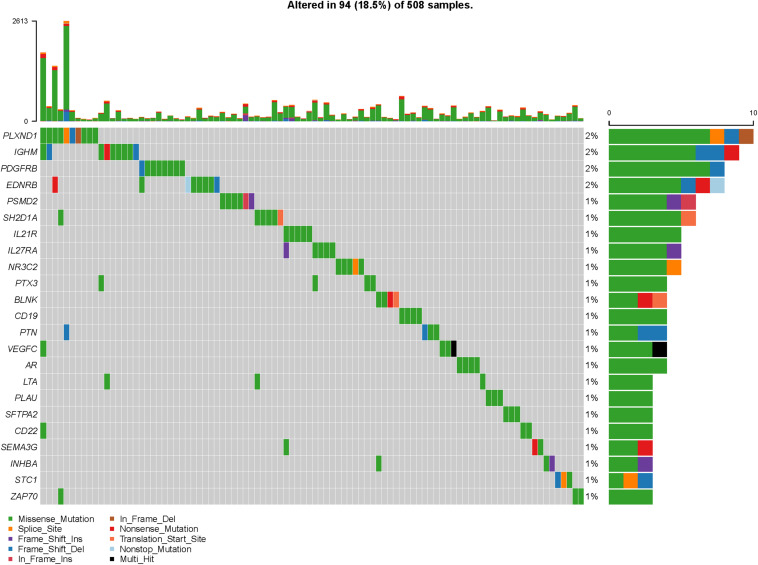
Mutations of core immune gene.

**FIGURE 5:**
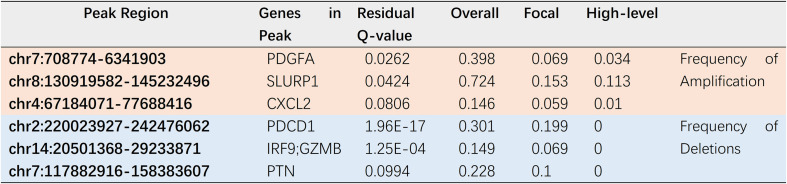
Copy number variation of hub immune genes.

To explore actively participating IRGs in the incidence and progression of HNSCC, we ascertained 64 IRGs that are differentially expressed and related to survival as hub IRGs (Table 1), including six chemokine-related genes, six interleukin (IL)-related genes, 12 growth factor-related genes, five protease-related genes, seven T- and B-cell surface molecule-related genes, six kinase-related genes, and 22 genes encoding other proteins. These IRGs showed excellent potential for monitoring prognostic biomarkers. A forest plot of hazard ratios (HRs) showed that most of these genes were protective factors (Figure 3A). PPI network analysis showed that some genes including colony-stimulating factor 2 (CSF2), chemokine receptors and their ligands (CXCR3, CXCR4), clusters of differentiation (CD22, CD79A), ILs (IL-1A, IL-1β), Granzyme B (GZMB), programmed cell death 1 (PDCD1), zeta chain-associated protein kinase 70 (ZAP70), transforming growth factor-β_3_ (TGF-β_3_), plasminogen activator urokinase (PLAU), tumor necrosis factor receptor superfamily 4, 12A, and 25 (TNFRSF4, 12A, and 25), and inducible costimulator (ICOS) were the hub IRGs among the dataset (Figure 3B). The clinical value of these hub IRGs is significant; therefore, it is important to comprehensively explore their molecular characteristics. These hub IRGs present genomic instability (mutations and copy number alterations) in tumors of HNSCC patients, and missense mutations are the most commonly occurring type (Figures 4, 5).

**TABLE 1:** The tumor-associated function of 56 core IRGs.

Types	No./Gene names	Functions	References
Chemokine-related genes	CXC motif chemokine receptor 4 (CXCR4)	Tumor proliferation, angiogenesis, invasion. Migration promoted through MMP-2/MMP-9 or MEK1/2 and ERK1/2 pathways	González-Arriagada et al., 2018
	CXC motif chemokine receptor 3 (CXCR3)	Induction of cytoskeletal remodeling and EMT through the AKT pathway, invasion, and metastasis of tongue squamous cell carcinoma	Li Z. et al., 2018
	CXC motif chemokine ligand 13 (CXCL13)	Promotes osteoclast activation and OSCC invasion	Sambandam et al., 2013
	CXC motif chemokine ligand 2 (CXCL2)	Affects the expression of CDK4, cell proliferation in esophageal cancer, Bone destruction	Wang et al., 2009
	CC motif chemokine ligand 26 (CCL26)	Binding to CCR3 receptors increases the expression of IL6 and IL8 and promotes tumor invasion	Sun et al., 2018
	CC motif chemokine receptor 8 (CCR8)	Regulates the function of T_reg_ and promotes tumor migration and invasion	Liu et al., 2019
Interleukin- related genes	Interleukin1β (IL1β)	The CCL22-CCR4-FOXp3 pathway is involved in tumor genesis and development	Li et al., 2019
	Interleukin 21 receptor (IL21R)	Affects the migration of cancer cells through the MMP pathway	León et al., 2015
	Interleukin1α (IL1α)	Participates in the inflammatory process	Li et al., 2019
	Interleukin 34 (IL34)	Promotes the differentiation of monocytes and macrophages, tumor growth, metastasis, and angiogenesis	Endo et al., 2020
	Interleukin 27 receptor subunit α (IL27Rα)	Mediates inflammatory response, T lymphocyte infiltration	Sénécal et al., 2016
	Interleukin 2 receptor subunit γ (IL2Rγ)	Regulates the differentiation of multiple lymphocyte lineages	Goh and Hong, 2017
Growth factor-related genes	Colony-stimulating factor 2 (CSF2)	GM-CSF stimulates HNSCC cell invasion and metastasis by upregulating MMP-2 and MMP-14 expression	Hong, 2016
	Lymphotoxin α (LTα)	Regulating the TNFR/NF-κB signaling pathway mediates PFKF33-dependent glycolysis and promotes tumor angiogenesis of HNSCC	Lauenborg et al., 2015
	TNF receptor superfamily member 12α (TNFRSF12α)	Stimulation of the NF-κB signaling pathway. As a prognostic marker for PTC	Qiu et al., 2018
	TNF receptor superfamily member 25 (TNFRSF25)	Enhanced T cell memory in patients with metastatic HNSCC. Stimulates NF-κB activity and regulates apoptosis	Schreiber et al., 2012
	TNF receptor superfamily member 4 (TNFRSF4)	Activating NF-κB promotes the expression of apoptosis inhibitors BCL2 and BCL2lL1/BCL2-XL, thereby inhibiting apoptosis	Schreiber et al., 2012
	Interferon regulatory factor 9 (IRF9)	The antiproliferative activity of IFN is mediated by the JAK–STAT pathway	Nan et al., 2018
	Inhibin subunit βA (INHβA)	The TGF-β/Smad pathway is activated to regulate EMT	Chen et al., 2019
	Transforming growth factor-β_3_ (TGF-β_3_)	The main inducer of EMT, promotes the growth and metastasis of HNSCC	Qin et al., 2016
	Platelet-derived growth factor receptor β (PDGFRβ)	Facilitates the rearrangement of actin cytoskeleton and proliferation of tumor cells	Song et al., 2017
	Endothelin receptor type β (EDNRβ)	Promotes the growth of tumor cells in tongue squamous cell carcinoma by MAPK pathway	Vasaikar et al., 2018
	Platelet-derived growth factor subunit A (PDGFA)	Promotes the proliferation and migration of mesenchymal cells	Song et al., 2017
	Vascular endothelial growth factor C (VEGFC)	Promotes angiogenesis and lymphangiogenesis, immune escape	Miao et al., 2018
Protease-related genes	Zeta chain of T cell receptor-associated protein kinase 70 (ZAP70)	Bcl-2 expression is upregulated by NF-κB and AKT pathways, promoting tumor metastasis	Gladkikh et al., 2017
	Granzyme B (GZMB)	CTL activation is also an important effector molecule of NK cytotoxicity	Mhaidat et al., 2014
	Plasminogen activator, urokinase (PLAU)	Promotes tumor migration and invasion	Magnussen et al., 2014
	Plasminogen activator, urokinase receptor (PLAUR)	Associated with poor prognosis of OSCC	Magnussen et al., 2014
	Proteasome 26S subunit, non-ATPase 2 (PSMD2)	The cell cycle of the G_0_/G_1_ phase is regulated by P21 and/or P27	Li Y. et al., 2018
T and B cell surface molecule-related genes	CD19	It forms a complex with CD21 that blocks the B cell receptor signaling pathway	Liu et al., 2020
	CD79A	Promotes tumor genesis and metastasis	Luger et al., 2013
	Programmed cell death 1 (PACD1)	Mediated immune escape	Ran and Yang, 2017
	CD22	CD19 signal transduction is inhibited by B cell receptors and co-receptors	Kim et al., 2020
	Inducible T cell costimulatory (ICOS)	Cell signaling, immune response, and cell proliferation	Fan et al., 2020
	SH2 domain-containing 1A (SH2D1A)	Mediates two-way stimulation of T cells and B cells	Koochakzadeh et al., 2015
	CD247	As a biomarker for PTC	Ye et al., 2019
Kinase-related genes	Gastrin (GAST)	Regulates autophagy through the STK11-Prkaa2-ULk1 pathway	Rao et al., 2017
	Gonadotropin-releasing hormone 1 (GNRH1)	Participates in the self-renewal and dry maintenance of lung cancer stem cell-like cells through upregulation of the JNK signaling pathway	Lu et al., 2015
	Stanniocalcin1 (STC1)	Promotes apoptosis by phosphorylation of P65 by PI3K/AKT, IκB and IKK signaling	Pan et al., 2017
	Stanniocalcin2 (STC2)	Promotes HNSCC migration by regulating PI3K/AKT/Snail signaling pathway	Xue et al., 2019
	Androgen receptor (AR)	A shorter CAG repeat length in the gene was associated with an adverse outcome in HNSCC	Rosa et al., 2007
	Nuclear receptor subfamily 3 group C member 2 (NR3C2)	Mediates the effect of aldosterone on salt and water balance in restricted target cells	Zhao et al., 2020
Others	Baculoviral IAP repeat-containing 5 (BIRC5)	Inhibits apoptosis and ensures proper chromosome separation	Frassanito et al., 2019
	Pentraxin 3 (PTX3)	Mediates maladjustment of mitotic signaling pathways and tumor escape	Chan et al., 2017
	Pleiotrophin (PTN)	Promotion of tumor proliferation and inhibition of apoptosis-reduced chemotherapy sensitivity	Zhou et al., 2018
	SHC adaptor protein 1 (SHC1)	The immunosuppressive effect of STAT3 was enhanced, and the immune surveillance effect of STAT1 was decreased in breast cancer	Ahn et al., 2017
	Retinol-binding protein 1 (RBP1)	Contributes to the uptake of retinol. Upregulation is associated with poor prognosis in TSCC	Chen et al., 2018
	Progestagen associated endometrial protein (PAEP)	PAEP/glycoprotein stimulates the TGF pathway and PKC cascade. Inhibits T lymphocyte activation, proliferation, and cytotoxicity	Weber et al., 2019
	Surfactant protein A2 (SFTPA2)	Enhances the phagocytosis and chemotaxis of alveolar macrophages	Maitra et al., 2010
	Dickkopf WNT signaling pathway inhibitor 1 (DKK1)	Inhibits WNT signaling and promotes proliferation, invasion, and growth in cancer cell lines	Sun et al., 2019
	Plexin D1 (PLXND1)	Mediates invasion and metastasis of prostate cancer cells through Notch-induced cell migration and regulation of E-cadherin	Vivekanadhan and Mukhopadhyay, 2019
	Semaphorin 3G (SEMA3G)	Inhibition of tumor cell migration and invasion	Zhou et al., 2012
	B cell linker (BLNK)	Inhibits lymphocyte differentiation in tumors, leading to disease progression	Lee et al., 2020
	Secreted LY6/PLAUR domain containing 1 (SLURP1)	Activates cholinergic transmission and promotes T cell development	Bergqvist et al., 2018
	Immunoglobulin heavy chain (including IGHM, IGHV12, IGHV3.64, and IGHV4.34)	IGH gene was significantly correlated with tumor recurrence rate. Different gene rearrangement affects the diversity of immunoglobulin	Thörnqvist and Ohlin, 2018
	T cell receptor α variable region (Including TRAV2, TRAV4, TRAV8.3, TRAV8.6, TRAV26.1, and TRBJ2.3)	TRAV-TRAJ gene recombination is associated with antigen recognition, and the diversity of TRAV genes provides more protective immunity	Pakasticali et al., 2019

*AKT, serine/threonine kinase; CDK4, cyclin dependent kinase 4; CTL, Cytotoxic T lymphocytes; ERK, Extracellular signal-regulated kinase; HNSCC, head and neck squamous cell carcinoma; IκB, inhibitor of nuclear factor kappa-B; IKK, inhibitor of nuclear factor kappa-B kinase; IRG, immune-related gene; JAK, Janus kinase; MAPK, mitogen-activated protein kinase; MEK, MAP kinase/ERK kinase 1; MMP, matrix metalloproteinase; NF, nuclear factor; NK, natural killer; OSCC, oral squamous cell carcinomas; PFKF, phosphofructokinase; PKC, protein kinase C; PTC, papillary thyroid carcinoma; PI3K, phosphoinositide 3-kinase; STAT, signal transducer and activator of transcription; TNF, tumor necrosis factor; TSCC, tongue squamous cell carcinoma.*

### Transcription Factor Regulatory Network

**FIGURE 6:**
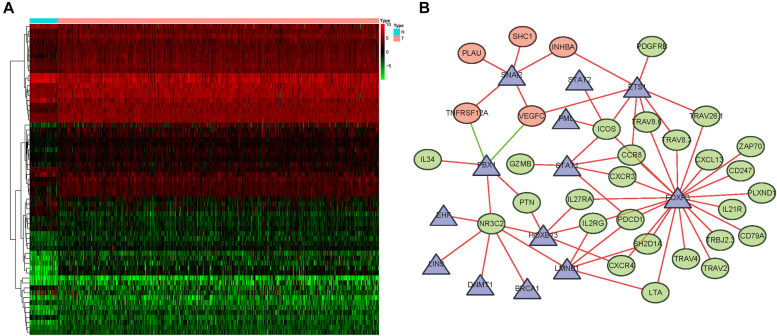
Regulatory networks mediated by transcription factors (TFs). **(A)** Differentially expressed TFs. **(B)** A network of tumor-related TFs that regulate the expression of core immune genes. Among them, the triangle represents TFs, the green circle represents core immune genes related to good prognosis, and the red circle represents immune genes related to poor prognosis. The red line represents positive regulation, and the green line represents negative regulation.

To investigate potential molecular mechanisms correlated with the clinical significance of the hub IRGs, we analyzed the regulatory mechanisms of these genes. We examined the expression profiles of 318 TFs and found that 63 of them were differentially expressed between HNSCC tissues and adjacent normal tissues (Figure 6A). We then built a regulatory network to establish the link between these differentially expressed TFs and 64 previously identified hub IRGs. Correlation scores (> 0.4) and *p*-value (< 0.001) were set as the cutoff thresholds. The TF-based regulatory network demonstrated the regulatory relationships between IRGs and TFs (Figure 6B).

### Evaluation of Clinical Outcomes

**FIGURE 7:**
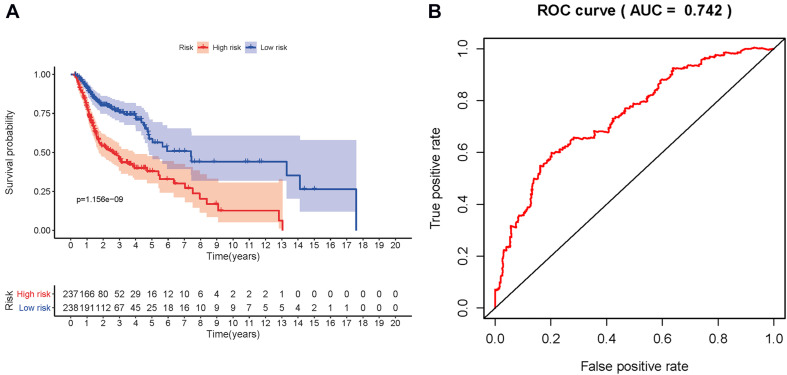
Construction of prognosis model. **(A)** Patients in the high-risk group had a shorter overall survival. **(B)** Receiver operating characteristic (ROC) curve verification of prognosis index.

**FIGURE 8:**
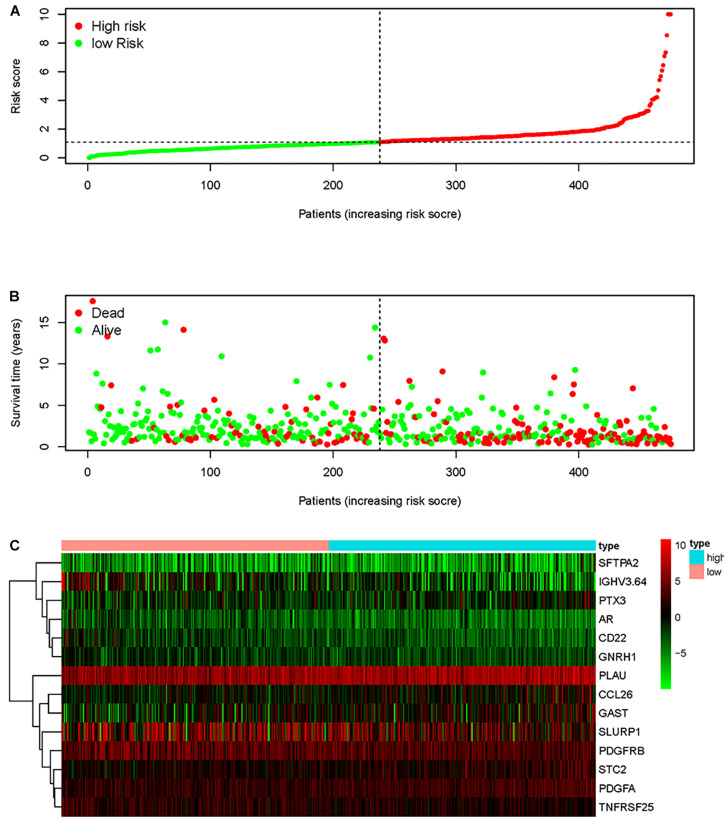
Prognostic indicators based on core immune genes. **(A)** Group and distribution of prognostic indicators. **(B)** Survival status of patients in different groups. **(C)** Thermogram of the core immune genes used to construct the model.

**FIGURE 9:**
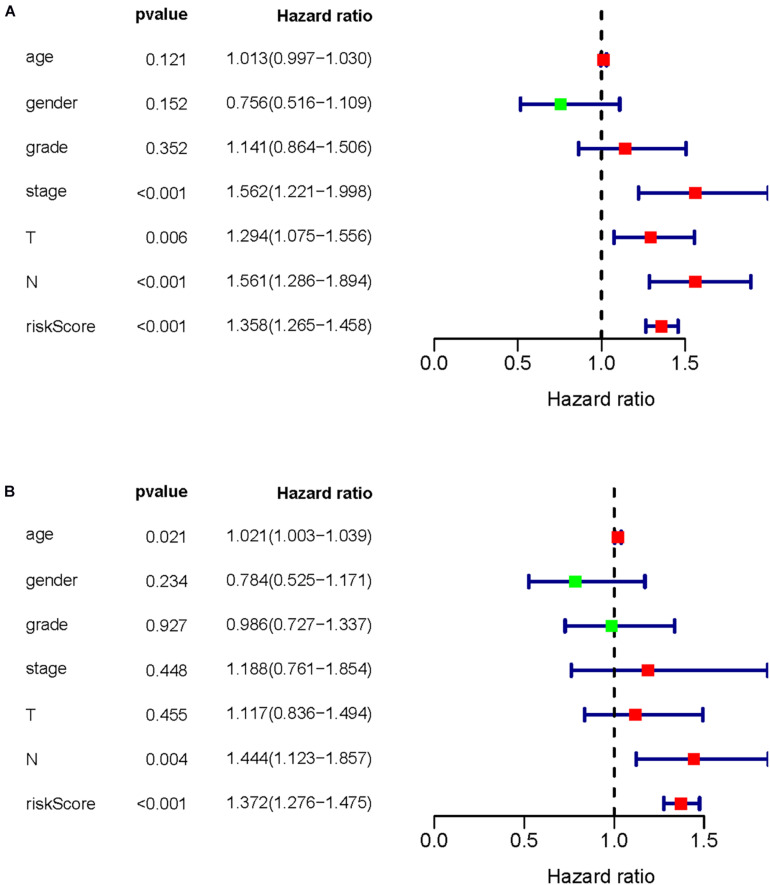
Prognostic value of common clinical parameters and prognostic indicators of core immune genes. **(A)** Univariate Cox analysis was used to evaluate the prognostic value of common clinical parameters and core immune genes. **(B)** Multivariate Cox analysis was used to evaluate the prognostic value of common clinical parameters and core immune genes.

A prognostic signature was built based on the results of LASSO Cox regression analysis to divide the HNSCC patients into high-risk and low-risk groups (Figure 7A). The formula was as follows: [expression level of PLAU × 0.0018] + [expression level of SFTPA2 × 0.0557] + [expression level of PTX3 × 0.0230] + [expression level of PDGFRB ×(−0.0209)] + [expression level of CCL26 × 0.0068] + [expression level of CD22 × (−0.0790) + [expression level of IGHV3-64 × (−0.0131)] + [expression level of GAST × 0.0163] + [expression level of GNRH1 × (−0.4339)] + [expression level of PDGFA × 0.0294] + [expression level of SLURP1 × (−0.0012)] + [expression level of STC2 × 0.0173] + [expression level of AR × (−0.2028)] + [expression level of TNFRSF25 × (−0.0561)]. This immune-based prognostic index could be an important tool for predicting HNSCC patient outcomes. The OS rates at 3 years for the high-risk group and the low-risk group were 45.4 and 75.6%, respectively. To examine the predictive accuracy of the model for OS, we used time-dependent ROC curves. The results showed that the AUC of the ROC curve was 0.742, which has moderate survival monitoring potential for the prognostic model based on hub IRGs (Figure 7B). The risk scores and survival status of each HNSCC patient are shown in Figures 8A,B. A heat map was generated to describe the expression patterns of the risk genes in the two prognostic groups (Figure 8C). More importantly, the prognostic signature became an independent predictor after adjustment for clinical parameters, including age, gender, tumor grade, tumor stage, lymph node metastasis status, and tumor size (Figure 9).

### Clinical Utility of the Prognostic Signature

**FIGURE 10:**
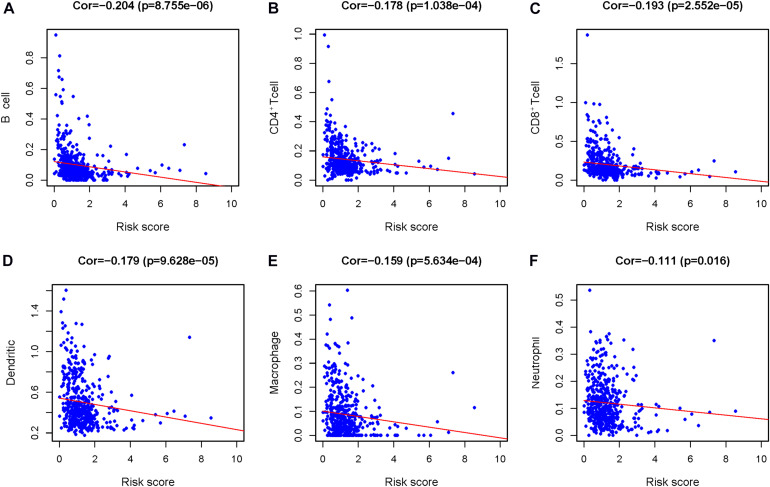
The relationship between the prognosis index of core immune gene and the infiltration amount of six types of immune cells. Correlation analysis was performed using Pearson test. **(A)** B cells, **(B)** CD4^+^ T cells, **(C)** CD8^+^ T cells, **(D)** dendritic cells (DCs), **(E)** macrophages, and **(F)** neutrophils.

To investigate whether our prognostic signature can reflect the state of HNSCC patients’ tumor immune microenvironment, we evaluated the components of tumor-infiltrating immune cells in HNSCC tissues and analyzed the correlation between the risk score and the fractions of tumor-infiltrating immune cells. With an increase in the risk score, the fractions of tumor-infiltrating immune cells (CD8^+^ T cells, B cells, neutrophils, macrophages, CD4^+^ T cells, and DCs) decreased (Figure 10).

## Discussion

It is well known that immune cells infiltrating the tumor microenvironment are considered to perform key roles in the biological behaviors of solid cancers, which are closely associated with clinical prognosis. Although the significance of IRGs in cancer generation and progression as well as immunotherapy has been proven, an integrative, genome-wide profiling model correlated to their clinical significance and molecular mechanisms is not well established. This comprehensive analysis of IRGs in HNSCC helps us to understand their clinical significance and underlying molecular characteristics. A large number of HNSCC tissue samples are available from TCGA database, which ensures that the results of this study are reliable enough.

The present study identified several IRGs significantly involved in the generation and progression of HNSCC that could serve as potential valuable clinical biomarkers. Moreover, the underlying molecular mechanisms were explored by bioinformatics analyses. Importantly, a selected differentially expressed IRG-based personalization immune prognostic signature was developed for defining immune cell infiltration, and its potential clinical application value was explored. Although there is a deeper understanding of tumorigenesis and tumor immunology, many aspects of HNSCC immune-related molecular mechanisms are not well elaborated. Immunologically, the malignant transformation of cells is closely related to chronic inflammation of the local microenvironment; therefore, this study was focused on hub genes (IRGs associated with prognosis). Several studies have found differentially expressed genes between HNSCC and non-tumor tissue samples (Saada-Bouzid et al., 2019), providing intrinsic insight into the pathogenesis of HNSCC at the genetic level. However, the characteristics of IRGs in HNSCC have not been comprehensively investigated to date. Here, we concentrated on HNSCC IRGs and signaling pathways by combining immunogenomic profiles with their corresponding clinical significance to describe the immune status of HNSCC more comprehensively.

Through univariate Cox analysis, we found that 236 IRGs were closely related to the OS of HNSCC patients, indicating that IRGs are very important for the prognosis of HNSCC patients. CCL26, SEMA3G, DKK1, GAST, GNRH1, PDGFA, and ZAP70 genes are related to the OS of HNSCC, which is consistent with the results of Li et al. (2020). Analysis of gene function enrichment showed that these prognoses related to immune genes were mainly involved in the interaction of cytokine–cytokine receptor interactions and the nuclear factor (NF)-κB signaling pathway. The interaction of cytokine receptors mainly involves the RAS–mitogen-activated protein kinase (MAPK) and Janus kinase (JAK)–signal transducer and activator of transcription (STAT) pathways. JAK–STAT signals control cell proliferation, differentiation, and survival by transferring external signals from the plasma membrane to the nucleus, thus participating in the occurrence, development, transfer, and drug resistance mechanism formation of HNSCC (Liu et al., 2016). The NF-κB signaling pathway is an effective regulator of many important physiological processes, including cell proliferation, apoptosis, angiogenesis, inflammation, and immune response (Monisha et al., 2017). In previous studies, the NF-κB pathway was found to usually be activated with the progression of HNSCC, and its persistent expression is the root cause of cancer cell proliferation, invasion, and metastasis and the low survival rate of HNSCC patients (Popeda et al., 2019). Our results suggest that the BIRC5 gene might be the target gene of NF-κB, which can inhibit cell apoptosis by binding p65 and BIRC5 (Yan et al., 2013; Zeng et al., 2016), leading to HNSCC diffusion and poor prognosis; however, this needs to be verified by subsequent experiments.

In addition, we identified 64 differentially expressed and prognosis-related IRGs as core genes, including six chemokine-related genes, six IL-related genes, 12 growth factor-related genes, five protease-related genes, seven T- and B-cell surface molecule-related genes, six kinase-related genes, and 22 genes encoding other proteins (Table 1). CXCR3 and CXCR4 are known to promote the proliferation, invasion, and migration of HNSCC through matrix metalloproteinase (MMP)-2/MMP-9 (González-Arriagada et al., 2018; Li Z. et al., 2018). CXCL2 and CXCL13 are associated with bone destruction in oral cancer (Wang et al., 2009; Sambandam et al., 2013) and promote tumor invasion. This is consistent with the results of our KEGG analysis. CCL26 and CCR8 are related to the Ca^2+^ mobilization of cells, and CCR8 can also recruit Treg infiltration, thereby promoting tumor metastasis (Sun et al., 2018; Liu et al., 2019). Genes related to Treg infiltration also include the IL-related gene IL-1β, which induces Treg infiltration through the CCL22–CCR4–Foxp3 pathway and participates in the development of HNSCC (Li et al., 2019). Other IL-related genes can promote tumor metastasis (León et al., 2015; Sénécal et al., 2016; Goh and Hong, 2017; Endo et al., 2020). Growth factor-related genes are also associated with tumor metastasis, among which LTα, TNFRSF12α, TNFRSF25, TNFRSF4, and IRF9 can mediate HNSCC tumorigenesis and metastasis through the NF-κB signaling pathway (Schreiber et al., 2012; Lauenborg et al., 2015; Nan et al., 2018; Qiu et al., 2018). The NF-κB pathway was one of the important pathways in the KEGG analysis of this study. The protease-related gene ZAP70 mediates prostate cancer metastasis through the NF-κB pathway (Gladkikh et al., 2017). Among the seven genes related to the surface molecules of T and B cells, CD19 and CD79A are associated with the development and activation of B cells (Luger et al., 2013; Liu et al., 2020). PACD1 encodes PD-1 and is normally expressed in T cells, but its expression in tumor tissues is closely related to tumor immune escape (Ran and Yang, 2017). The expression of six kinds of kinase-related genes is related to the poor prognosis of the tumor. Among them, more research has been conducted on the anti-cisplatin effect of GAST (Rao et al., 2017), and GNRH1 and STC1 can activate the c-Jun N-terminal kinase (JNK) pathway to facilitate tumor development (Lu et al., 2015; Pan et al., 2017). Studies have shown that BIRC5 may be the target gene of NF-κB in genes encoding other proteins, and the expression of its apoptosis inhibitor survivin affects chromosome separation (Frassanito et al., 2019). PTX3 and PTN also affect the prognosis of tumors *via* the NF-κB pathway (Chan et al., 2017; Zhou et al., 2018). In addition to the NF-κB pathway, another important pathway, namely, the RAS/MAPK pathway, was identified in the KEGG analysis. Some studies have shown that SHC1 downregulates immune surveillance through the RAS/MAPK pathway to promote tumor development (Ahn et al., 2017). In summary, the core IRGs involve tumor-related pathways including the phosphoinositide 3-kinase (PI3K)/AKT, NF-κB, Notch, Wnt, JAK/STAT, TGF-β/Smad, PKC, and RAS/MAPK pathways. The 5 growth factor-related genes (LTα, TNFRSF12α, TNFRSF25, TNFRSF4, and IRF9) and BIRC5, PTX3, and PTN all play key roles in the occurrence and development of HNSCC mediated by the NF-κB pathway.

To explore the regulatory mechanism of the abnormal expression of IRGs in HNSCC, we constructed a regulatory network of core IRGs and tumor-related TFs. The results showed that FOXP3, STAT1, STAT2, SNAI2, and EHF play core roles in the network. Foxp3 regulates the differentiation and function of Tregs and coregulates Treg maturation with T-cell receptor (TCR) signaling (Ono, 2020). Core gene analysis showed that TCR diversity was determined by six genes in the TRAV family. Second, Foxp3 can also inhibit NF-κB transcriptional activity with P65 in TSCC (Li K. et al., 2018), contributing to the infiltration of Tregs in tumors. Previous studies have shown that the maturation of Tregs is related to TNFRSF in core genes (Schreiber et al., 2012), which indicates that Tregs play an important role in the development of HNSCC. STAT1 and STAT2 regulate interferon genes. The heterodimers of STAT1 and STAT2 enter the nucleus together with IRF9 and transcriptionally activate IFN-1. Studies have shown that STAT1 is a TF downstream of cytokines and growth factors. Reduced phosphorylation also inhibits the expression of class I antigen-processing element (APM), allowing HNSCC to evade CTL killing (Ryan et al., 2020). Phosphorylated STAT2 induces the proliferation of oral cancer cells (Hao et al., 2019). Both SNAI2 and EHF can regulate genes related to epithelial cells. Wang et al. (2019) showed that SNAI2, as a DNA-binding TF, can promote the initiation of EMT induced by TGF-β, resulting in decreased intrinsic cell adhesion and enhanced motor ability, which is conducive to its proliferation, migration, and invasion. EHF is an epithelial-specific TF that plays an important role in maintaining normal cell homeostasis and mediating epithelial tissue differentiation. Its ectopic expression in ESCC promotes cell proliferation and invasion (Wang et al., 2019). PML, PBX1, and DNMT1 act on DNA, and PML and DNMT1 participate in DNA modification and activate/silence genes through demethylation/methylation (Gajewski et al., 2013). PBX1 enhances DNA binding and plays a role in the carcinogenesis of ESCC. In the TF network analysis, the TFs Foxp3 and SNAI2 were found to participate in regulating the expression and silencing of some core genes, providing some references for research on the genesis and development mechanism of HNSCC.

It is worth mentioning that we used univariate Cox to analyze the prognosis based on IRGs and then used LASSO Cox analysis to build the prognostic model. The results show that this model can well distinguish patients with different clinical results. We further evaluated its reliability by ROC curve analysis (the AUC of the ROC curve was 0.742). Compared with Bhattacharya et al. (2014), who only used Cox regression to establish a prognosis model for thyroid cancer, after we added LASSO regression based on Cox regression, many genes with unique values were found to control the complexity of the model through the parameter λ to avoid overfitting. Moreover, the prognostic markers we constructed can be used as independent predictors after clinical parameter correction, yielding high clinical applicability for the prediction of HNSCC development. The establishment of an HNSCC prognostic model based on IRGs provides a reference for clinical treatment, which is not only helpful to assess patient condition but also helpful to further elucidate the functions of IRGs.

The theory of tumor immune escape suggests that the process of tumor generation and development can be divided into three stages: elimination, equilibrium, and escape. Tumor-infiltrating immune cells play an important role in this process. It is known that tumor-infiltrating immune cells are prognostic factors for different types of cancer, and their number and type can reflect the type of immune response in the tumor microenvironment, which is heterogeneous (Balermpas et al., 2016). Monisha et al. (2017) compared the infiltration of immune cells in normal and HNSCC tissues with CIBERSORT and discussed its clinical value (Monisha et al., 2017). They found that CD4^+^ memory T cells were related to the prognosis of HNSCC. Therefore, the Pearson test was utilized to analyze the relationship between the prognostic index and the infiltration quantity of six kinds of immune cells (CD8^+^ T cells, B cells, neutrophils, macrophages, CD4^+^ T cells, and DCs) in this study. The results showed that with an increase in the risk value, the number of tumor-infiltrating immune cells decreased. Our prognostic index can reflect the immune microenvironment of HNSCC patients to a certain extent. Previous studies have shown that CD8^+^ T cells combine with MHC-I molecules to promote the cytotoxic effect of TILs in HNSCC patients, and the survival rate of patients with high CD8 expression is improved (Andreu et al., 2010). At present, the role of B cells in the tumor microenvironment is still controversial. Tumor-infiltrating B cells (TIL-B) can act as local APCs and provide critical secondary costimulation signals to tumor-infiltrating CD8^+^ T cells to maintain the antitumor ability mediated by CD8^+^ T cells and prolong the survival time of patients (Andreu et al., 2010). Gao et al. (2018) found that activation of FcγR by B lymphocytes can recruit suppressive leukocytes, thus inhibiting the antitumor immune response and promoting the progression of tumors. Tumor-associated macrophages (TAMs) can be divided into M_1_ (which can produce Th_1_ cytokines) and M_2_ (which secretes IL-10 and other immunosuppressive cytokines) subtypes. M_1_ TAMs phagocytized HNSCC cells in a CD47-dependent manner. In the study by Gao et al. (2018), HNSCC cells cocultured with monocytes were found to transform monocytes into M_2_ macrophages. M_2_ macrophage-released EGF induces epithelial–mesenchymal transformation (EMT) in HNSCC cells, which promotes the migration and invasion of cancer cells (Cho et al., 2018). It has been found that increased neutrophils in the peripheral blood can lead to a poor prognosis and local infiltration and distant metastasis in HNSCC patients. Cho et al. (2018) also confirmed that neutrophils are the main sources of CCL4 and MMP9 in the tumor microenvironment, and cytokines released by neutrophils can promote the adhesion and migration of the HNSCC cell matrix; namely, the increase in neutrophils can promote the progression of HNSCC. CD4^+^ T cells are stimulated by different cytokines and are transformed into corresponding helper T cells. Some studies have shown that CD4^+^ CD69^+^ T cells are associated with a good prognosis in HNSCC patients, while FOXP3^+^ CD4^+^ T cells are favorable for tumor progression (DeNardo et al., 2009). DCs are the most powerful APCs known and play a key role in immune responses against cancer. According to Long et al. (2019), the depletion of DCs in tumor tissue indicates a high recurrence rate and poor prognosis of HNSCC, while high infiltration of DCs is related to a better prognosis. The lack of DCs in HNSCC may lead to insufficient stimulation of cytotoxic T lymphocytes and the formation of Tregs, leading to tumor immune tolerance (Wischatta et al., 2000), while the presence of DCs might activate the immune monitoring system, facilitate ingestion and presentation of tumor antigens, and induce an antitumor immune response.

However, there are a few limitations in this study. First, we only used the expression data and clinical information of the HNSCC queue in TCGA database, and we should also use other databases [such as the Gene Expression Omnibus (GEO) database] for verification. Second, the molecular mechanisms of these IRGs involved in the occurrence and development of HNSCC need to be further studied by *in vitro* and *in vivo* experiments. Finally, the AUC of ROC was 0.742, suggesting that the model has moderate potential for the prognostic signature based on IRGs in survival monitoring. The model could divide patients into the high-risk group and low-risk group and predict the survival outcomes of HNSCC patients. However, it is not high enough for clinical practice. We will collect clinic samples of HNSCC patients in the next period to optimize the model and improve the value of AUC.

In conclusion, we explored the core IRGs that may play important roles in the development of HNSCC and further revealed the potential regulatory mechanisms of these core IRGs. In addition, we constructed an ideal prognosis model through LASSO Cox. These results are helpful for developing individualized treatment plans and new treatment targets for HNSCC patients.

## Data Availability Statement

Publicly available datasets were analyzed in this study. This data can be found here: https://cancergenome.nih.gov/; http://portals.broadinstitute.org/; and https://string-db.org/.

## Author Contributions

JL, SCZ, YO, FJ, and ZZ contributed to the conception and design. JL and SCZ contributed to the collection and assembly of data. JL, SCZ, XZ, ZH, YW, and YY contributed to the data analysis and interpretation. JL, SCZ, XZ, and ZZ contributed to the manuscript writing. ZZ, WW, and FJ contributed to the manuscript revision. All authors gave the final approval for the manuscript.

## Conflict of Interest

The authors declare that the research was conducted in the absence of any commercial or financial relationships that could be construed as a potential conflict of interest.

## Footnotes

1 https://cancergenome.nih.gov/

2 http://portals.broadinstitute.org/

3 https://string-db.org/
